# ProInterVal: Validation of Protein–Protein Interfaces through Learned Interface Representations

**DOI:** 10.1021/acs.jcim.3c01788

**Published:** 2024-03-25

**Authors:** Damla Ovek, Ozlem Keskin, Attila Gursoy

**Affiliations:** † KUIS AI Center, Koç University, Istanbul 34450, Turkey; ‡ Computer Engineering, Koç University, Istanbul 34450, Turkey; ¶ Chemical and Biological Engineering, Koç University, Istanbul 34450, Turkey

## Abstract

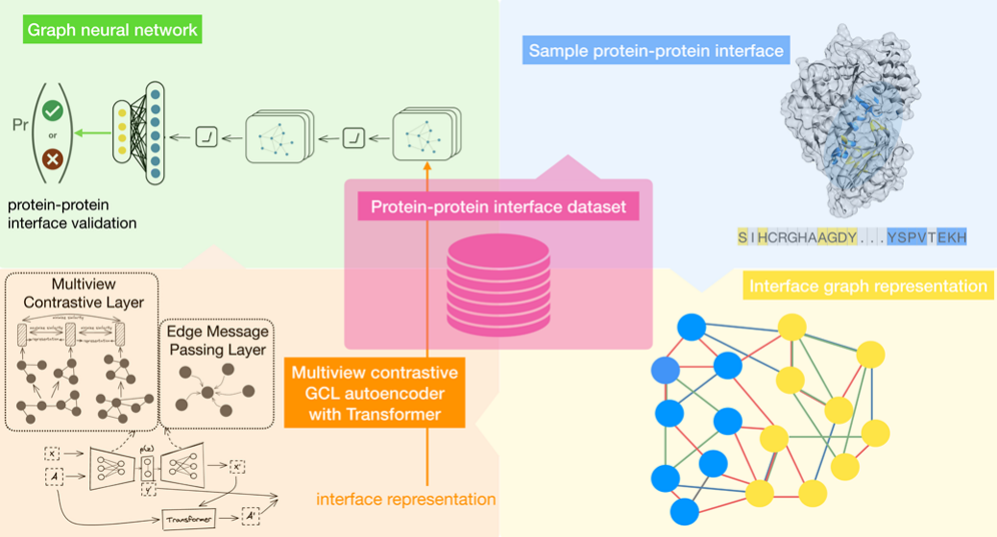

Proteins are vital components of the biological world and serve a multitude of functions. They interact with other molecules through their interfaces and participate in crucial cellular processes. Disruption of these interactions can have negative effects on organisms, highlighting the importance of studying protein–protein interfaces for developing targeted therapies for diseases. Therefore, the development of a reliable method for investigating protein–protein interactions is of paramount importance. In this work, we present an approach for validating protein–protein interfaces using learned interface representations. The approach involves using a graph-based contrastive autoencoder architecture and a transformer to learn representations of protein–protein interaction interfaces from unlabeled data and then validating them through learned representations with a graph neural network. Our method achieves an accuracy of 0.91 for the test set, outperforming existing GNN-based methods. We demonstrate the effectiveness of our approach on a benchmark data set and show that it provides a promising solution for validating protein–protein interfaces.

## Introduction

Proteins perform essential functions in the cells by interacting with other proteins. These versatile molecules have diverse functions, including catalyzing chemical reactions, transporting molecules, offering structural support, participating in signaling pathways, and enabling ion transport.^1^ Proteins interact with each other through protein interfaces (binding sites). These interfaces greatly enhance our understanding of biological functions.^2^ Any abnormal interactions may disrupt cellular functions, causing detrimental effects on the organism. Investigating protein–protein interfaces is of significant importance in disease treatment and pharmaceutical research.

In computational studies, proteins are typically represented by their sequence and/or structure. Three-dimensional structures capture the arrangement of atoms and provide details about functional sites and interaction surfaces. Deep learning approaches are employed to analyze and extract meaningful features from these 3D structures and the primary sequences of proteins. Recurrent neural networks (RNNs)^3^ are utilized to capture sequential dependencies among residues in the primary sequences. Convolutional neural networks (CNNs)^4−6^ are used to learn hierarchical representations by applying filters to the local regions of the protein structures. DeepInterface,^7^ DOVE,^8^ and DeepRank^9^ are CNN models that examine the protein–protein interfaces using 3D voxels. However, CNNs are not rotation invariant and require data augmentation to mitigate orientation sensitivity, which affects the computational performance during training. Recently, graph neural networks (GNNs) have gained popularity, with the representation of protein–protein interfaces as rotation-invariant graphs allowing GNNs to overcome these limitations.^10,11^ GNN-based studies model proteins as graphs, where nodes represent amino acids or atoms, and edges denote spatial, sequential, and/or functional relationships.^10,11^ For example, GNN-DOVE^11^ employs a GNN model, representing atoms’ chemical properties as node features and interatom distances as edge features. Similarly, DeepRank-GNN^10^ is a GNN-based approach that scores docking models by converting interface residues into two graphs: one representing internal edges between residues from the same chain and another representing external edges between residues from different chains.

With the advancements in graph-based deep learning methods, graph-based protein representation learning has also gained considerable attention due to its ability to capture the complex interactions and structural dependencies at protein–protein interfaces. By leveraging multiview contrastive learning and self-prediction tasks, essential geometric features of proteins are effectively captured.^12−14^ Transformers are also used to investigate protein–protein interfaces and differentiate native-like protein complexes from incorrect conformations by combining Vision Transformer^15^ architecture with hybrid and multiattention networks.^16^

Most existing studies are constrained by the varying degree of limited expressivity and complexity of manual feature calculations and require more extensive training data sets to cover a large amount of structural information and further improve accuracy.

This study presents a graph-based approach, called ProInterVal, for validating the biological relevance of protein–protein interfaces using learned interface representations with the help of an extensive training data set. Our approach combines a novel graph contrastive autoencoder and a transformer to learn representations, and then validates interfaces with a GNN model. As in a previous study,^12^ our model incorporates a multiview contrastive layer to capture the similarity between correlated substructures within proteins and an edge message passing layer to capture the interdependence among different interactions involving a residue and its neighboring residues. ProInterVal distinguishes itself from other compared models by capitalizing on learned representations of protein–protein interfaces. Employing a few handcrafted features is limited to generating the input graph for representation learning, as the validation of protein–protein interfaces depends solely on the learned representation. Our method exhibits a test set accuracy of 0.91, surpassing the performance of the current GNN-based approaches.

## Data and Methods

In this section, we delve into the architecture of our model, which combines a graph-based contrastive autoencoder with a transformer for learning an adequate representation of protein–protein interfaces. The input to our model is a protein complex comprising two chains. Initially, we extracted the interface and converted it into a graphical representation. We then processed this graph by using a graph-based contrastive autoencoder combined with a transformer architecture to acquire a latent representation of the graph. Finally, based on the learned representation, we utilized a graph neural network (GNN) model to generate an interaction score for the two chains. Through this multistep process, our model effectively validates protein–protein interactions.

### Data Set for Interface Representation Learning

We employed multiple data sources for training and testing of our model. We utilized a large set of unlabeled protein–protein complexes for training and testing our graph-based contrastive autoencoder combined with a transformer for generating protein representations. These complexes were obtained from the Protein Data Bank (PDB).^17^ The data set comprises a total of 534,203 samples.^18^ PDB and chain IDs of the samples are listed in the Supporting Information and can be downloaded from PDB and served on request.

By leveraging this extensive data set, we aimed to capture a diverse range of protein–protein interactions and their corresponding interfaces. The availability of a sizable unlabeled data set allowed our model to learn robust representations without being limited by labeled data. In the subsequent sections, we describe how this data was processed and used in training our graph-based framework for investigating protein–protein interfaces.

### Data set for Protein–Protein Interface Validation

#### DeepInterface Data Set

The DeepInterface data set^7^ served as a crucial component for the validation of protein–protein interactions. This data set was obtained from four sources: DOCKGROUND,^19^ PPI4DOCK,^20^ PIFACE,^21^ and PDB.^17^

Positive interfaces were primarily obtained from PIFACE, which contains interface structures extracted from the PDB files published until 2012. Additional positive interfaces were extracted from the PDB files deposited after 2012 to avoid redundancy. Negative interfaces were derived from DOCKGROUND and PPI4DOCK decoy sets, using CAPRI’s classification.^22^ In the context of our work, a positive interface refers to the interface found in a protein–protein complex that exists in the Protein Data Bank (PDB) (assuming that it is biologically relevant). Conversely, a negative interface corresponds to the interface observed in a docked protein–protein complex with an unacceptable Critical Assessment of Predicted Interaction (CAPRI) score, meaning that it has a fraction of native contact (Fnat) value lower than 0.1, ligand root-mean-square deviation (LRMSD) greater than 10 Å, and interface root-mean-square deviation (iRMSD) greater than 4 Å.

Table 1 showcases the quantitative distribution of samples from each source, emphasizing their contribution to the data set and the partitioning scheme into distinct subsets for training, validation, and test purposes.

**Table 1:** Distribution of Positive and Negative Protein Interface Data from Diverse Sources within the DeepInterface Dataset, Illustrating the Partitioning Scheme into Distinct Subsets for Training, Validation, and Test Purposes

	PIFACE	PPI4DOCK	DOCKGROUND	PDB2012	TOTAL
training	127,790	127,790			255,580
validation			4632	4632	9264
test			4632	4632	9264
TOTAL	127,790	127,790	9264	9264	274,108

To ensure a fair comparison, we evaluated our model alongside two graph-based docking model evaluation models, namely, GNN-DOVE and DeepRank-GNN. The evaluation was performed on three data sets: DeepInterface data set, GNN-DOVE data set, and DeepRank-GNN data set.

#### GNN-DOVE Data Set

GNN-DOVE utilizes a combination of the Dockground data set^19^ and ZDOCK^23^ as well as a CAPRI scoring data set.^24^ To eliminate redundancy, the complexes were grouped based on sequence alignment and TM align.^25^ Specifically, two complexes were assigned to the same group if any pair of proteins from the two complexes exhibited a TM score greater than 0.5 and a sequence identity of 30% or higher. Dockground and ZRANK are used for training and validation, while the CAPRI scoring data set is designated for testing purposes.

#### DeepRank-GNN Data Set

The DeepRank-GNN data set is composed of the Docking Benchmark version 5 (BM5).^26^ The BM5 comprises a nonredundant set of 142 complexes (dimers), excluding antibody–antigen complexes and complexes with more than two chains. A total of 25,300 models per complex were generated using the integrative modeling software HADDOCK.^27^ The test set consisted of all docking models generated for 15 randomly selected complexes (379,500 models, 10% of the data set), while the remaining 127 complexes were split into a training set (80%, 102 complexes) and a validation set (20%, 25 complexes).

### Protein–Protein Interface Representation Learning

#### Input Preparation and Graph Construction

In our study, the initial step involves the extraction of the protein–protein interface within the protein complexes. In a complex, each partner has a protein interaction (binding) site, where some residues are directly in contact with the other partner’s interaction site, while neighboring residues, though not in direct contact, still play a role in the binding process. To extract the interface, we employ a procedure to identify the residues that interact with each other. Precisely, we assess the proximity of residues from different chains by comparing their distances concerning the sum of their van der Waals radii and an additional threshold of 0.5 Å. If the distance between two residues satisfies this criterion, we consider them to be contacting residues. Once all of the contacting residues are identified, we define the neighboring residues. Neighboring residues are determined as those within a distance of 6 Å from any contacting residue within the same chain. Consequently, the union of the contacting and neighboring residues defines the interface region of the protein complex.

Subsequently, we represent the interface region as a graph, wherein the residues are represented as nodes and their interactions are captured through edges. Each node is encoded as a vector of length 30, encompassing the following features: residue type, polarity, residue charge, relative accessible surface area (relASA) in the monomer and in the complex form, knowledge-based pair potential (PP), and backbone dihedral angles (ϕ and ψ). Naccess is used to calculate relASA,^28^ and knowledge-based PP are obtained from Keskin et al.^29^

Naccess has various hyperparameters such as z-slices, probe size, van der Waals value file, and parameters specifying whether to ignore HETATM records, hydrogens, and water.^28^ In this study, we used default parameter values while running Naccess.

As in previous research of Zang et al.,^12^ we use three distinct edge types in our graph representation:

- Sequential edges: We establish an edge between the *i*th and *j*th residues if their sequential distance, denoted as |*j* – *i*|, falls below a predefined threshold. Specifically, an edge is created when |*j* – *i*| < 3, signifying proximity within the sequence.
- Radius edges: Besides sequential edges, we introduce edges between two nodes, i and j, if the Euclidean distance between them is less than a specified threshold of 10 Å. This criterion ensures that residues in close physical proximity are interconnected.
- K-nearest neighbor edges: Recognizing the potential variations in spatial scales across different proteins, we incorporate edges by connecting each node to its k-nearest neighbors based on the Euclidean distance. We set *k* = 10. This strategy ensures a comparable density of spatial edges across diverse protein graphs, facilitating meaningful graph representations.

The detailed descriptions of the node and edge features can be found in Supporting Table 1. The size of the graph and, hence, the adjacency matrix vary according to the size of the input protein–protein interface. However, each node and each edge are represented by fixed-size vectors of lengths 30 and 3, respectively. The embedding dimension is, on the other hand, fixed to 128 × 512. The input and output sizes of each layer in the architecture are summarized in Supporting Table 2.

**Figure 1:**
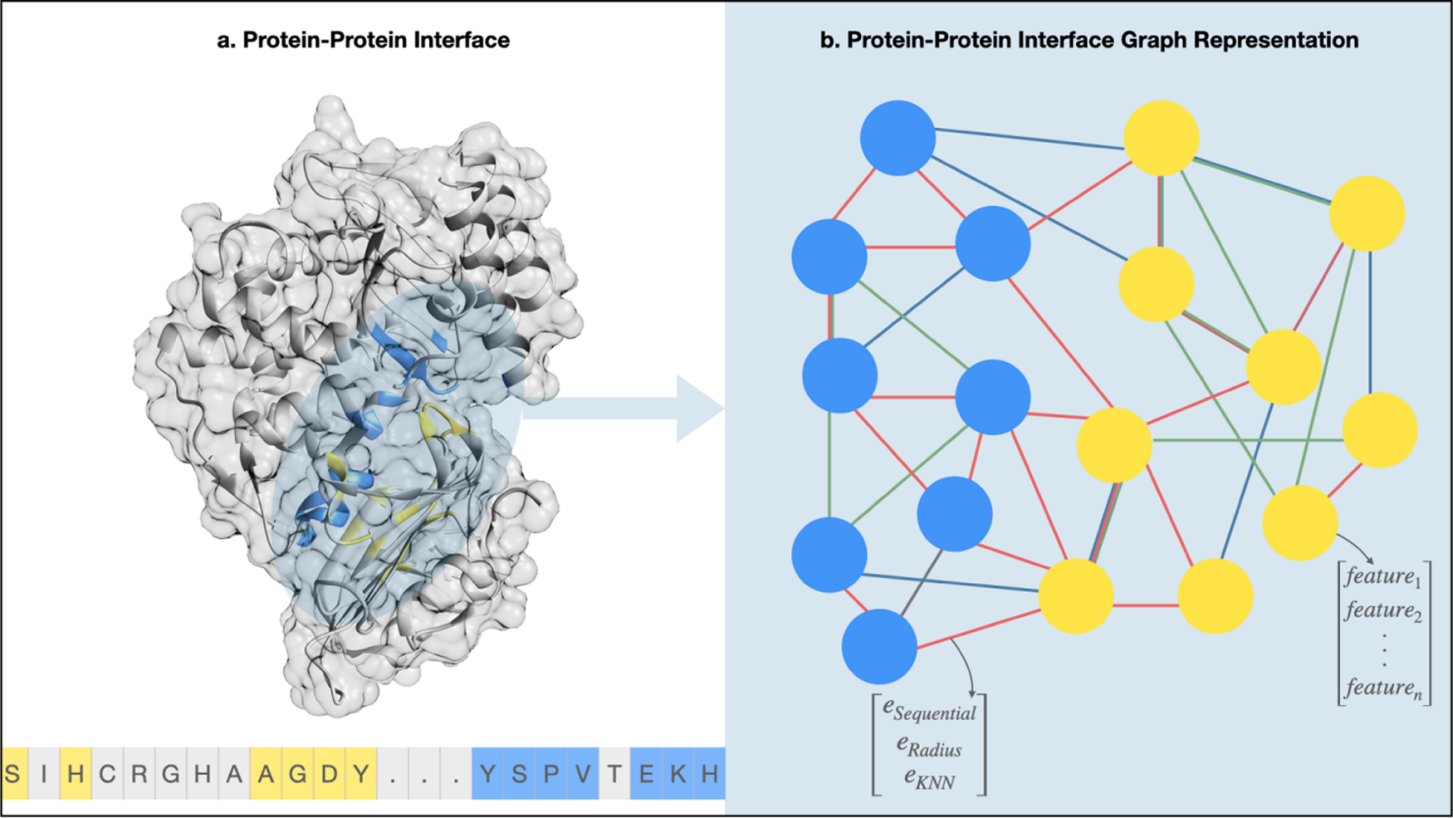
Protein–protein interface structure, sequence, and graph representation. (a) Visualization of a protein–protein complex (PDB ID: 1JTG_A_B) in a three-dimensional (3D) structure, depicted as a surface and ribbon model. The sequence of the complex is displayed at the bottom, with the interface residues highlighted in color. Residues from chain A are shown in blue, while residues from chain B are depicted in yellow. (b) Corresponding graph representation of the complex. The graph consists of nodes, each representing a residue and its associated features, and edges, representing different types of interactions (red: radius edges, green: sequence edges, and blue: KNN edges).

By employing these strategies, our graph-based approach enables comprehensive and informative encoding of the interface region, capturing sequential and spatial relationships between residues within the protein complex. Figure 1 shows the 3D structure and sequence of a protein–protein complex (interface residues are highlighted) and its graph representation. The nodes are represented as tensors, encompassing residue-specific features, while the edges are encoded as vectors of 1s and 0s, indicating the presence or absence of specific edge types.

#### Graph-Based Contrastive Autoencoder

**Figure 2:**
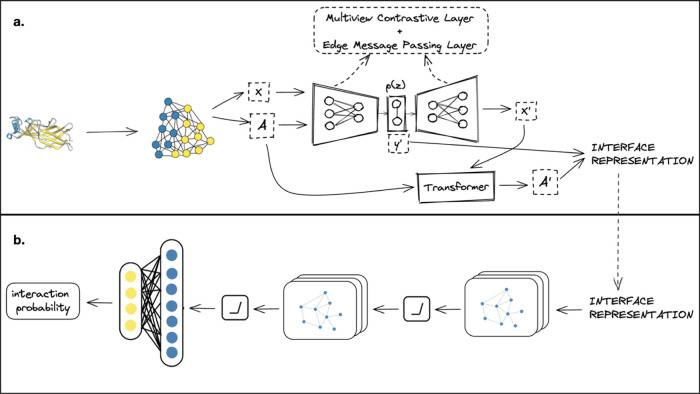
Overall framework. (a) Protein–protein interface structure is represented as a residue-level graph. The graph is input into a graph-based autoencoder combined with a transformer to learn the interface representation. (b) Learned representation is then passed through the GNN model to obtain the interaction probability.

After construction of the graph, it is passed through a novel graph autoencoder architecture combined with a transformer module to obtain a latent representation. Our proposed model is visually depicted in Figure 2a. The encoder component inputs the node feature matrix *X* and the adjacency feature tensor *A*, effectively encoding them into a continuous latent representation denoted as *z*. Conversely, given a point in the latent space, the decoder generates an output node feature matrix *X*′. Additionally, utilizing a disentangled latent space, the model predicts graph-level properties, yielding a graph property prediction denoted as *y*′.

#### Multiview Contrastive Layer

A multiview contrastive learning layer was added to the autoencoder to enhance the model’s performance. We employed the same approach taken by Zhang et al.^12^ The multiview contrastive learning involved the application of two cropping functions, namely subsequence and subspace.

(1) A consecutive protein segment was randomly sampled in the subsequence cropping, and the corresponding subgraph was extracted.
(2) In subspace cropping, a center residue was first sampled, and then all residues within a specified distance threshold were selected to form the subgraph.

Furthermore, a random transformation function was applied to the output subgraph, with options including identity (no transformation) and random edge masking (random removal of a fixed ratio of edges from the graph).

#### Edge Message Passing Layer

We added a message-passing layer to enhance the model performance further, as in a prior study.^12^ In message passing, a relational graph, commonly called a line graph, establishes connections among edges. Each node in the line graph represents an edge from the original graph. Specifically, edge (*i*, *j*, *r*_1_) in the original graph is linked to edge (*w*, *k*, *r*_2_) in the line graph only if *j* = *w* and *i* ≠ *k*. Subsequently, a relational graph convolutional network is applied to the line graph, enabling the derivation of the message function for each edge. The message function of an edge is updated by aggregating the features from its adjacent edges.

#### Transformer

The proposed model incorporates a transformer component for edge prediction in graph-based architectures inspired by the study of Mitton et al.^30^ The transformer takes the adjacency feature tensor and the predicted node feature matrix as inputs and generates a predicted edge feature tensor. The utilized transformer architecture follows a vanilla implementation.^31^

The vanilla transformer architecture is a neural network framework, initially designed for natural language processing tasks. At its core, it relies on a multihead self-attention mechanism, which allows the model to dynamically weigh the importance of different input elements. It consists of an encoder-decoder structure with both components comprising identical layers. Each layer contains two main sublayers: a multihead self-attention mechanism and a position-wise feedforward neural network that processes the attended representations, thus enabling the transformer to capture complex relationships and patterns in the data.

During the generation phase, the transformer sequentially adds edges to a graph. At each step, the input to the transformer consists of the node encoding of adjacent nodes to the predicted edge and the previously predicted edges in the graph. This iterative process allows the model to progressively construct the graph by making informed edge predictions based on the available information.

The model effectively captures the dependencies and interactions between nodes in a graph by employing a transformer-based approach, enabling accurate edge predictions. The iterative generation of edges ensures that the model incorporates the evolving graph structure while maintaining consistency with the existing edges in the graph.

The objective function of the transformer model can be expressed as
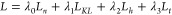
1where λ_*i*_ represents scaling constants with the values λ_0_ = 0.2, λ_1_ = 0.3, λ_2_ = 0.3, and λ_3_ = 0.2; *L*_*n*_ denotes the cross-entropy loss for reconstructing node features, *L*_*KL*_ corresponds to the *KL* divergence loss to calculate the statistical distance between the input graph and the latent space representation, *L*_*h*_ represents the mean squared error loss for predicting graph properties from the latent space, and *L*_*t*_ represents the cross-entropy loss for reconstructing edge features. The Supporting Information explains the methodology used to calculate lambda values.

To optimize the model, an Adam optimizer is employed with an initial learning rate of 5e^–4^.

### Protein Function Analysis of Learned Representations

*t*-SNE is a technique used for reducing the dimensionality of data in order to visualize the similarities among high-dimensional data points. To analyze the learned protein interface representations, *t*-SNE was employed to project them onto a two-dimensional (2D) space. A random sample set of 10,000 samples was selected, representing five function classes: antibodies, enzymes, receptors, hormones, and transporters. Within this sample set, there were 1879 antibodies, 849 enzymes, 1572 receptors, 2012 hormones, and 3,688 transporters. A t-SNE plot was generated to visualize the clustering patterns of these classes.

### Protein–Protein Interface Validation

Once the learned representation of two given proteins is obtained, a graph neural network model is employed to predict the interaction score between the given proteins. The GNN model consists of graph convolution layers (GCL), nonlinear activation functions (in this case, rectified linear units or ReLU), and pooling layers. The last layer of the model is fully connected. This architectural design enables the model to capture and process the structural information encoded in a graph representation. The output of this GNN model is a probability, which indicates the likelihood of the given protein complex being a biologically valid interaction.

Cross-entropy loss was used as the loss function to train the model, and the Adam optimizer was used for parameter optimization. The model was trained for 20 epochs with a batch size of 32.

## Results and Discussion

### Ablation Study

To assess the individual contributions of specific components in our model, we conducted ablation experiments with a PPI validation task. We systematically removed key elements, namely, the multiview contrastive layer and the edge message passing layer. Then, we trained each model with the DeepInterface training set and compared the prediction accuracy of the test set. This rigorous analysis allowed us to evaluate the impact of each component on the overall performance of the model and gain insights into its respective roles in the learning process. As shown in Table 2, the results can be significantly improved by using multiview contrastive learning, and the prediction performance further increases after performing edge message passing.

**Table 2:** Ablation Experiment Results of Graph Autoencoder, Multiview Contrastive Layer, and Edge Message Passing Layer on the PPI Validation Task

model	accuracy
graph autoencoder	0.764
multiview contrastive graph autoencoder	0.888
graph autoencoder with edge message passing	0.879
ProInterVal	0.923

#### Multiview Contrastive Layer

The multiview contrastive layer is designed to capture the similarity between correlated protein substructures by constructing informative views of the substructures. The layer’s objective is to preserve the similarity between these substructures both before and after mapping them to a latent space. By creating multiple views that reflect different aspects of the protein substructures, the multiview contrastive layer enables the model to capture and preserve the intricate relationships and similarities among the substructures. This contributes to the overall effectiveness of the model in capturing the complex nature of the protein interactions and representations.

#### Edge Message Passing Layer

The edge message passing layer can be regarded as a modified version of the pair representation update specifically tailored for graph neural networks. Drawing inspiration from AlphaFold2,^32^ which utilizes the triangle attention mechanism in transformers to capture pair representations, our edge message passing layer aims to capture the interdependence among various interactions involving a residue and its sequentially or spatially adjacent residues. Unlike triangle attention in AlphaFold2, our approach incorporates angular information to effectively model diverse types of edge interactions, enabling more efficient sparse edge message passing operations.

As stated in the Data and Methods section, we adopted our multiview contrastive and edge message passing layers from Zhang et al.^12^ However, our method has the following differences: they apply a contrastive autoencoder to the graph of the whole proteins, but we extract and encode only the graph of the interface regions of the protein complexes; the task is different such that they used learned representations to identify protein function and fold classification, whereas we used them to validate protein–protein interfaces in the docking complexes. Moreover, our model involves a transformer component that they do not have and a GCL-based autoencoder, which is different from their autoencoder architecture.

### PPI Validation Performance Evaluation

**Figure 3:**
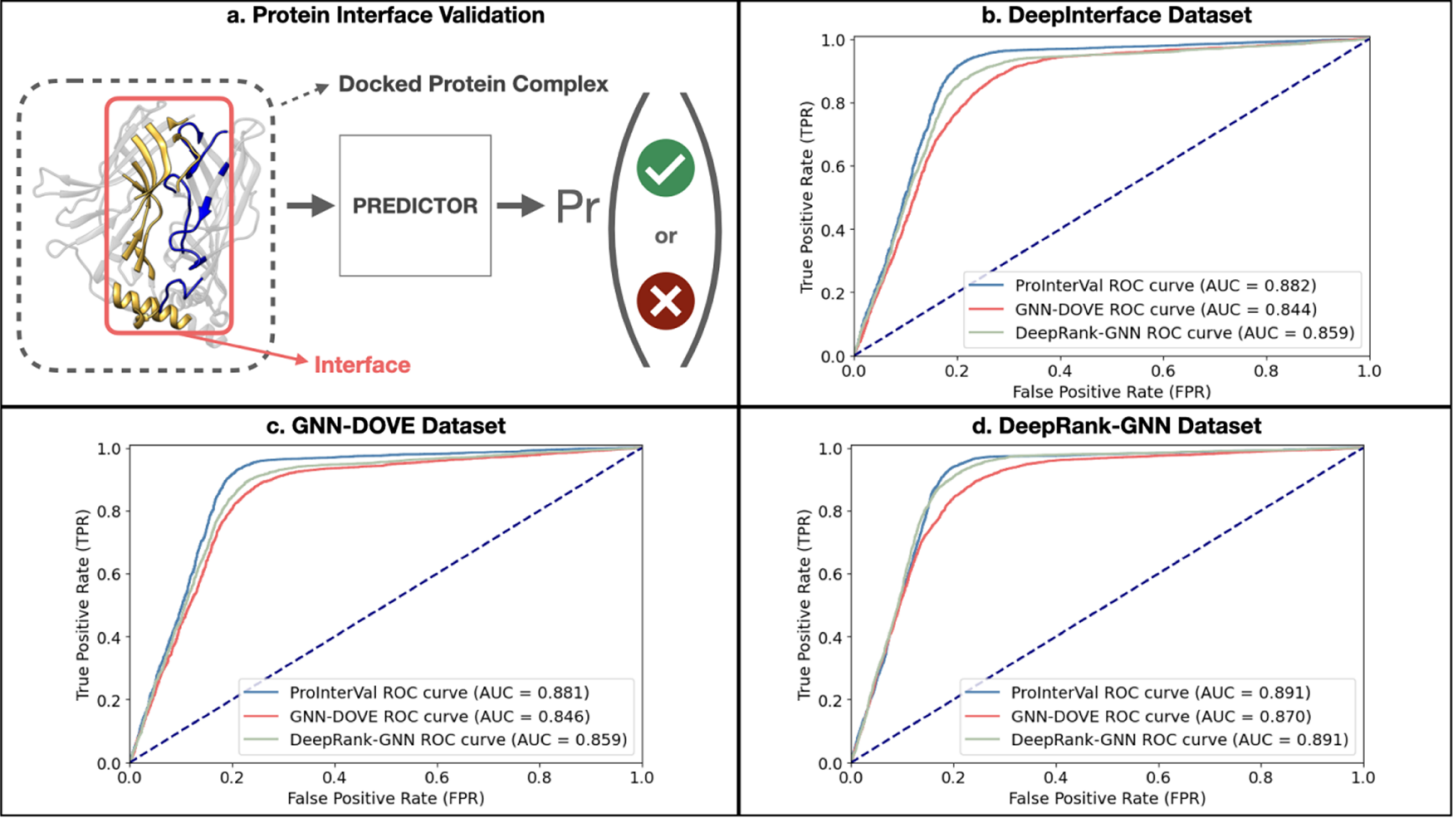
Protein–protein interface validation and performance comparison of ProInterVal, GNN-DOVE, and DeepRank-GNN on multiple data sets. (a) Illustration depicting the concept of protein–protein interface validation, where the predictor generates a probability for the docked complex interface to be a biological complex. (b) Performance evaluation results of ProInterVal, GNN-DOVE, and DeepRank-GNN on the DeepInterface test set after retraining the models from scratch on the training set of the DeepInterface data set. (c) Performance evaluation results of ProInterVal, GNN-DOVE, and DeepRank-GNN on the GNN-DOVE test set after retraining the models from scratch on the training set of the GNN-DOVE data set. (d) Performance evaluation results of ProInterVal, GNN-DOVE, and DeepRank-GNN on the DeepRank-GNN test set after retraining the models from scratch on the training set of the DeepRank-GNN data set.

We conducted a comprehensive performance comparison of our ProInterVal with the two state-of-the-art methods, GNN-DOVE and DeepRank-GNN, for validating protein–protein interactions. To ensure a fair evaluation, all models were trained from scratch on three distinct training sets and evaluated on their respective test sets. The details of these sets are explained in the data set section. Notably, the analysis revealed that ProInterVal consistently outperformed the other models across all three data sets. Figure 3 visually depicts the performance of ProInterVal compared to GNN-DOVE and DeepRank-GNN on these data sets. These findings highlight the robustness and efficacy of the ProInterVal model in accurately validating PPIs, underscoring its potential as a powerful tool in computational protein studies. Recently, a study based on 2D images of proteins has been published.^16^ Different from our approach, it is not a graph-based method and has an ROC-AUC score of 0.81 on the CAPRI score data set, while our method achieves an ROC-AUC score of 0.88 on the DeepInterface data set, constructed based on CAPRI classification, and the GNN-DOVE data set, sourced from the CAPRI score data set.

ProInterVal distinguishes itself from the compared models by leveraging learned representations of protein–protein interfaces, whereas the other models rely on handcrafted features to represent these interfaces. Protein function is governed by a wide range of structural motifs, characterized by intricate spatiochemical arrangements of atoms and amino acids. Handcrafted features struggle to comprehensively capture these patterns. Comparative modeling faces challenges in detecting these motifs, as the set of sequence/conformational perturbations that preserves function remains unknown. Therefore, this study introduces an approach that utilizes learned interface representations for validating protein–protein interfaces. Furthermore, as outlined in the Data and Methods section, our model is trained on a data set of over 500,000 interfaces, enabling it to capture diverse information and exhibit generalizability.

#### *t*-SNE Analysis

**Figure 4:**
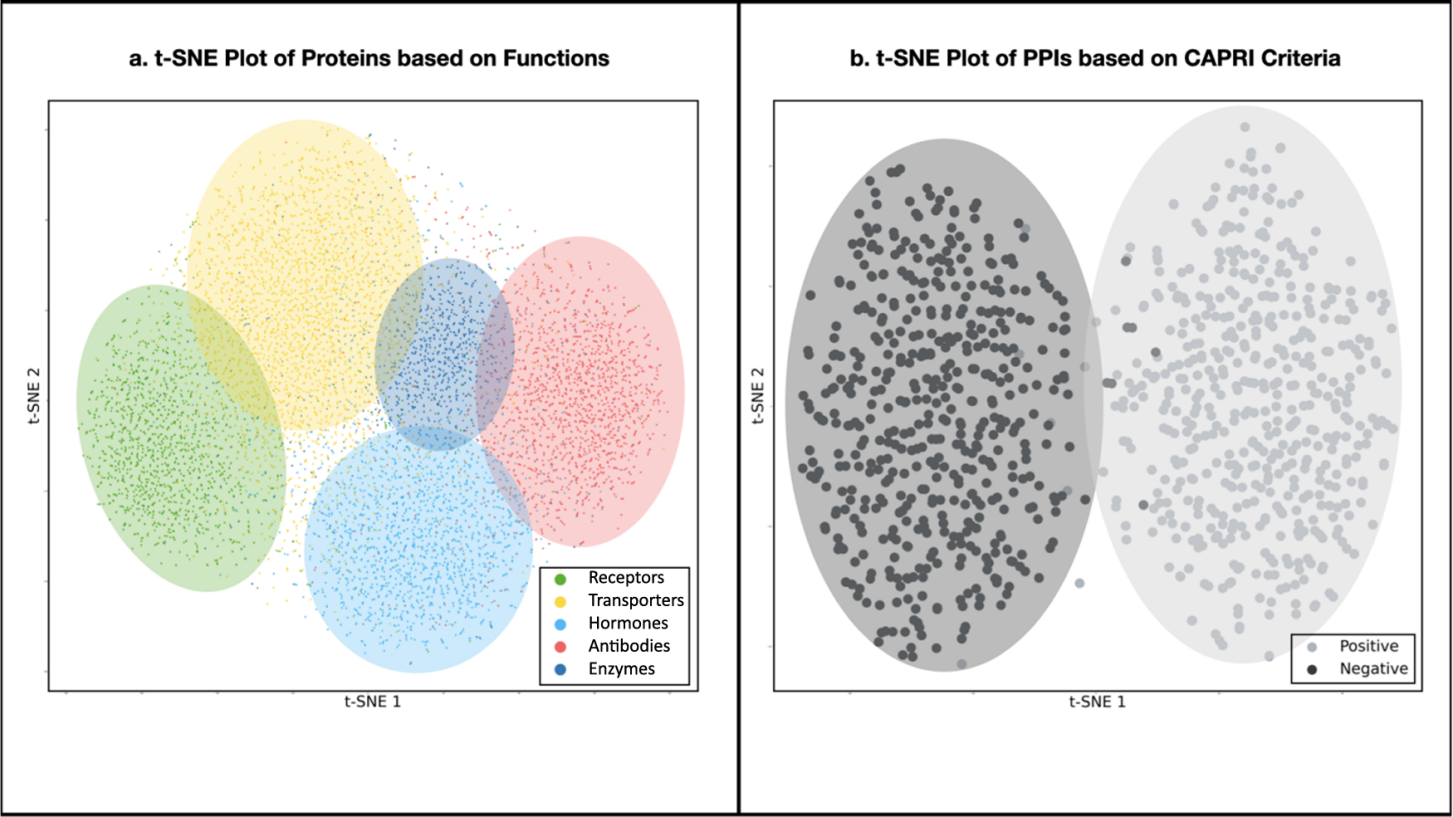
*t*-SNE graphs of representations learned by ProInterVal. (a) *t*-SNE plot, representing the clustering of protein functions based on learned representations. Each point corresponds to a protein and is color-coded according to its functional category. (b) *t*-SNE plot, illustrating the distribution of positive and negative protein–protein interfaces.

Figure 4 showcases the t-SNE visualizations of protein function clusters and positive/negative interfaces, offering valuable insights into the arrangement of protein functions and the differentiation between positive and negative interfaces. These visualizations demonstrate the potential of our approach in comprehending protein interactions and verifying their biological significance. As depicted in Figure 4a, the proteins exhibit clustering based on their functional classes in the embedding space, indicating that our model has captured functional fingerprints of the proteins to some extent.

In order to conduct a more in-depth analysis of the learned representations of interfaces in our model, a subset of 500 positive and 500 negative interfaces was randomly selected based on the CAPRI criteria. A *t*-SNE graph was then plotted to visualize the distribution and clustering patterns of these interfaces. The results of this analysis are presented in Figure 4.b. As can be seen from the figure, the positive and negative interfaces appear distinctly separated in the embedding space, indicating that our model has effectively learned a robust representation for the validation of protein–protein interactions.

### Ranking of the Positive Interfaces

In order to comprehensively evaluate of our model, we conducted a ranking analysis to assess its performance in distinguishing a single native interface (positive sample) from a set of approximately 100 incorrect docking models (negative samples). This evaluation involved comparing the performance of our model with GNN-DOVE and DeepRank-GNN as well as the docking models’ scoring function ZRANK.^33^

We randomly selected 100 positive protein–protein complexes from our DeepInterface test data set to carry out this analysis. For each of these 100 complexes, we generated 300 decoys using the GRAMM-X protein docking tool.^34^ From the pool of generated decoys, we carefully selected 100 incorrect decoys per complex based on the CAPRI criteria. As a result, we obtain a data set consisting of 100 incorrect decoys and one native structure per complex.

**Figure 5:**
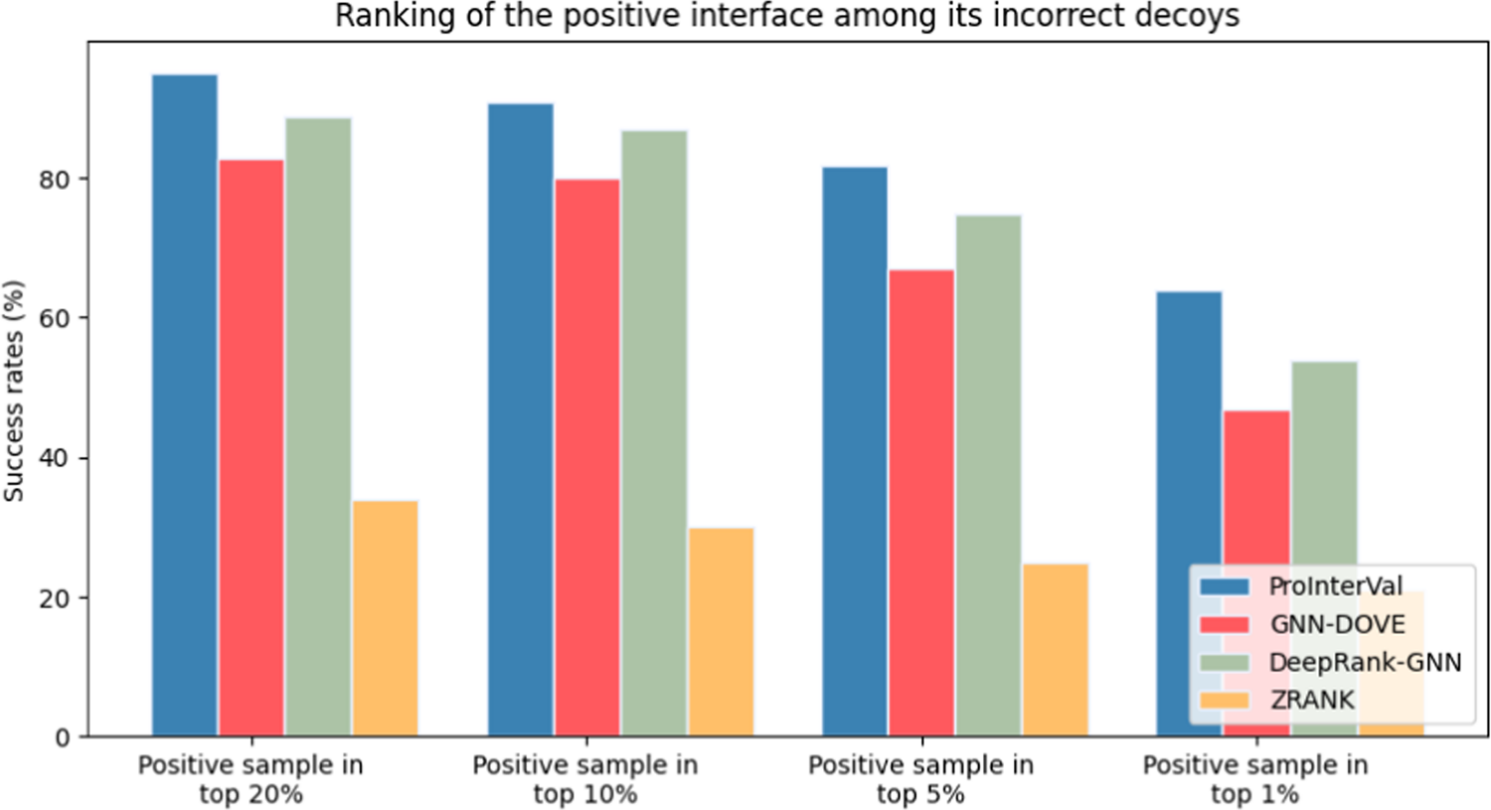
Ranking of the positive interface among the incorrect decoys of the corresponding complex.

Next, we applied ProInterVal, ZRANK, GNN-DOVE, and DeepRank-GNN to score and rank these structures. Specifically, we assessed the performance of these scoring tools by examining the position at which the native PDB structure (positive interface) was ranked among the 100 incorrect decoys (negative interface). When we refer to the “top 10%”, it signifies that the near-native interface is positioned within the uppermost 10% of its respective ranked list. Figure 5 shows the ranking results. Our model achieved a 63% success rate in the top 1 and a 90% success rate in the top 10%, and outperformed the others. In practical terms, this indicates that our model placed the native structure among the top 1% for 63 out of the selected 100 complexes and within the top 10% for 90 of these 100 complexes.

The remarkable performance of our model in interface ranking highlights the potential of integrating it into template-based protein–protein interaction (PPI) predictors to improve their overall predictive capabilities.

### Biological vs Crystal Interface Classification

X-ray crystallography has long been the predominant experimental method for determining the 3D structure of proteins, and it remains the most widely used technique since 2022. While X-ray structures are generally reliable and of high quality, erroneous structures are not uncommon. Common errors include misfitting of residues into electron density maps and the presence of synthetic oligomers resulting from crystal packing. The latter can lead to misleading conclusions and can impede accurate research findings. Therefore, it is crucial to develop tools to annotate crystallographic dimers as reliable biological or nonbiological (crystal) interfaces.

In this section, we assess the performance of ProInterVal in discriminating between biological and crystal interfaces using a DC data set. Additionally, we compare ProInterVal’s performance with that of DeepRank-GNN.

To train and evaluate ProInterVal, we utilized a data set comprising 5739 dimers from the MANY data set. The training set comprised 80% of the dimers, while the remaining 20% of the dimers constituted the validation set. The network was trained for 50 epochs, and the model with the lowest loss in the validation set was selected as the final model. Subsequently, this model was tested on the DC data set, which consists of 80 biological interfaces and 81 crystal interfaces with similar interface areas.

ProInterVal achieved approximately 88% accuracy, 88% precision, and an 85% F1 score on the test set. The results are shown in Table 3.

**Table 3:** Biological vs Crystal Interface Classification Performance Evaluation

	accuracy	precision	specificity	sensitivity	F1 score
ProInterVal	0.878	0.880	0.811	0.827	0.853
DeepRank-GNN	0.810	0.820	0.792	0.803	0.811

## Conclusions

In this research, we introduced an innovative approach for validating protein–protein interfaces by leveraging the learned representations. Our approach utilized a graph-based contrastive autoencoder architecture and a transformer to learn representations of protein–protein interaction interfaces from unlabeled data. This approach allowed us to capture the essential features of the interfaces and encode them into informative representations. The learned representations were then validated by using a graph neural network (GNN) model designed for PPI validation.

The results of our experiments on a benchmark data set demonstrated the effectiveness of our proposed method. We achieved an accuracy of 0.91 for the test set, surpassing the performance of the existing GNN-based methods. To the best of our knowledge, GNN-DOVE and DeepRank-GNN are the state-of-the-art GNN-based methods. Therefore, a comparison of our proposed method with theirs shows the superiority of our work. Our results highlight the potential of the learned representations in accurately validating protein–protein interactions.

Furthermore, the learned representations offer versatility in various downstream tasks. For instance, they can be used for the classification of protein complexes as either crystal or biological, helping researchers discern the validity and reliability of crystallographic dimers.

Integrating our model into a template-based PPI predictor can potentially enhance the performance of existing prediction methods. By incorporation of the learned representations, the template-based predictor can leverage the rich information encoded in the representations, resulting in more accurate and reliable predictions.

In a future work, incorporating sequence information into structural information can further augment the effectiveness of the representation learning model. By integration of these complementary data sources, the model can capture a more comprehensive view of protein–protein interactions, considering both the structural characteristics of the interfaces and the sequence patterns involved.

## Data Availability

The detailed information about the data sets is available in the Supporting Information and they can be obtained directly from PDB (https://www.rcsb.org/), and the source code is available on https://github.com/ku-cosbi/ProInterVal.

## Supporting Information Available

For a comprehensive understanding of our methodology, the Supporting Information provides detailed explanations of the data retrieval process, input graph’s node and edge feature specifications, and comprehensive information about each component’s inputs, outputs, and hyperparameters in our framework. Furthermore, additional results and findings are presented (PDF)
