# Supplementary material for: ProInterVal: Validation of Protein–Protein Interfaces through Learned Interface Representations
Source: J Chem Inf Model. 2024 Mar 25;64(8):2979–87. doi: 10.1021/acs.jcim.3c01788 (PMC11040718; doi:10.1021/acs.jcim.3c01788)
Supplement: Supplementary file 1 — ci3c01788_si_001.pdf [file ci3c01788_si_001.pdf]

Supporting Information

ProInterVal: Validation of Protein-Protein Interfaces through Learned Interface Representations

Damla Ovek,[†,‡] Ozlem Keskin,[¶] and Attila Gursoy[*,‡]

[†]KUIS AI Center, Koç University, Istanbul, Turkey

[‡]Computer Engineering, Koç University, Istanbul, 34450, Turkey

[¶]Chemical and Biological Engineering, Koç University, Istanbul, 34450, Turkey

*To whom correspondence should be addressed

E-mail: agursoy@ku.edu.tr

GitHub Repository: https://github.com/ku-cosbi/ProInterVal

Data Retrieval

1. **Protein-protein interface dataset for protein representation learning**

In this work, we used 534,203 protein-protein interfaces collected from PDB. 80% of the data is used for training and the rest (20%) is used for testing. The data set is taken from Abali et. al. and available at https://interactome.ku.edu.tr:8443/PPInt/. Additionally, the PDB and chain IDs of the samples in the dataset are provided in our GitHub repository.

2. **DeepInterface dataset**

To train and test the protein-protein interface validation component of our overall architecture, we used DeepInterface dataset. As explained in the paper, positive data comes from PIFACE and PDB deposited between 2012 and 2018, while negative data comes from DOCKGROUND and PPI4DOCK. The PDB and chain IDs of positive samples as well as model names of negative decoys, and their division as training, validation and test are available in in our GitHub repository.

3. **Other datasets used for comparisons**

In order to compare our model with the existing models, we have used two datasets that we called DeepRank-GNN dataset and GNN-DOVE dataset. DeepRank-GNN dataset is available from https://data.sbgrid.org/dataset/843/ [1]. MANY and DC datasets used for the classification of biological and crystal interfaces can also be found there. GNN-DOVE dataset is comprised of Dockground docking dataset, ZDOCK dataset, and CAPRI score set. All of them are downloadable from http://dockground.compbio.ku.edu/downloads/unbound/decoy/decoys1.0.zip, https://zlab.umassmed.edu/zdock/decoys_bm4_zd3.0.2_6deg.tar.gz, and http://cb.iri.univ-lille1.fr/Users/lensink/Score_set respectively.

Methodology Details

The objective function of the transformer model can be expressed as:

$$L = \lambda 0Ln + \lambda 1LKL + \lambda 2Lh + \lambda 3Lt$$

where λi represents scaling constants with the values λ0 = 0.2, λ1 = 0.3, λ2 = 0.3, and λ3 = 0.2; Ln denotes the cross entropy loss for reconstructing node features, LKL corresponds to the KL divergence loss to calculate the statistical distance between the input graph and the latent space representation, Lh represents the mean squared error loss for predicting graph properties from the latent space, and Lt represents the cross entropy loss for reconstructing edge features.

Therefore, the KL divergence loss is between the input graph and the latent space representation and lambdas are λ0 = 0.2, λ1 = 0.3, λ2 = 0.3, and λ3 = 0.2

We have started with the values λ0 = λ1 = λ2 = λ3 =0.25. As we try to balance multiple objectives in our loss function, i.e., cross entropy loss for reconstructing node features, KL divergence loss to calculate the statistical distance between the input graph and the latent space representation, the mean squared error loss for predicting graph properties from the latent space, and the cross entropy loss for reconstructing edge features, we normalize lambda values to sum up to 1. Then, we have tried different combinations of lambda values within a specified range and evaluated their impact on model performance. Our model performs best with the values λ0 = 0.2, λ1 = 0.3, λ2 = 0.3, and λ3 = 0.2.

Table S1: Descriptions of node and edge features.

Attribute	Description	Dimension
Node		
Residue Type	Twenty different residues (amino acids)	20
Polarity	Polar or non-polar	2
Charge	Positively charged, negatively charged, or neutral	3
relMonASA	Relative accessible surface area in the monomer form	1
relCompASA	Relative accessible surface area in the complex form	1
Pair Potential	Knowledge-based pair potential	1
Phi (φ)	Backbone dihedral angle	1
Psi (ψ)	Backbone dihedral angle	1
Edge		

Radius Edge	Closeness of two residues in Euclidean space (<10 Å)	1
Sequential Edge	Closeness of two residues in the protein sequence (<3)	1
KNN Edge	K-nearest neighbors of a residue in Euclidean space (k=10)	1

Table S2: Summary of the inputs, hyperparameters and outputs of each architecture component.

Model	Layer	Input size	Output size	#Parameters
**GCN Autoencoder**	Input (Node size)	30	-	-
	GCN Layer	30	64	64*30+64+64
	GCN Layer	64	32	64*32+32+32
	GCN Layer	32	16	32*16+16+16
	GCN Layer	16	128	16*128+128+128
	Multiview Contrastive	30	128	30*128+128
	Edge Message Passing	30	30	30*30+30
	GCN Layer	128	16	128*16+16+16
	GCN Layer	16	32	16*32+32+32
	GCN Layer	32	64	32*64+64+64
	GCN Layer	64	30	64*30+30+30
**Transformer**	Input	30	-	-
	Embedding Layer	30	128	30*128+128+128
	Multi-Head Self-Attention	128	128	4*(128*128+128+128)
	Feed-Forward Network	128	128	2*(128*128+128+128)
**GNN**	Input	128	-	-
	GCL Layer	128	64	128*64+64
	ReLU	64	64	-
	Max pooling	64	32	-
	FC Layer	32	2	128*2+2

Additional Results

We conducted a comprehensive performance comparison of our ProInterVal, with the two state-of-the-art methods, GNN-DOVE, and DeepRank-GNN, for validating protein-protein interactions. To ensure a fair evaluation, all models were trained on three distinct training sets and evaluated on their respective test sets. The results are evaluated based on the following metrics:

$$Accuracy = \frac{\mathrm{TP} + \mathrm{TN}}{\mathrm{TP} + \mathrm{TN} + \mathrm{FP} + \mathrm{FN}}$$

$$Sensitivity\ (Recall) = \frac{\mathrm{TP}}{\mathrm{TP} + \mathrm{FN}}$$

$$Specificity = \frac{\mathrm{TN}}{\mathrm{TN} + \mathrm{FP}}$$

$$Precision = \frac{\mathrm{TP}}{\mathrm{TP} + \mathrm{FP}}$$

$$MCC = \frac{\mathrm{TP} \times \mathrm{TN} - \mathrm{FP} \times \mathrm{FN}}{\sqrt{(TP + FP) \times (TP + FN) \times (TN + FP) \times (TN + FN)}}$$

where TP is the number of true positives, TN is the number of true negatives, FP is the number of false positives, and FN is the number of false negatives.

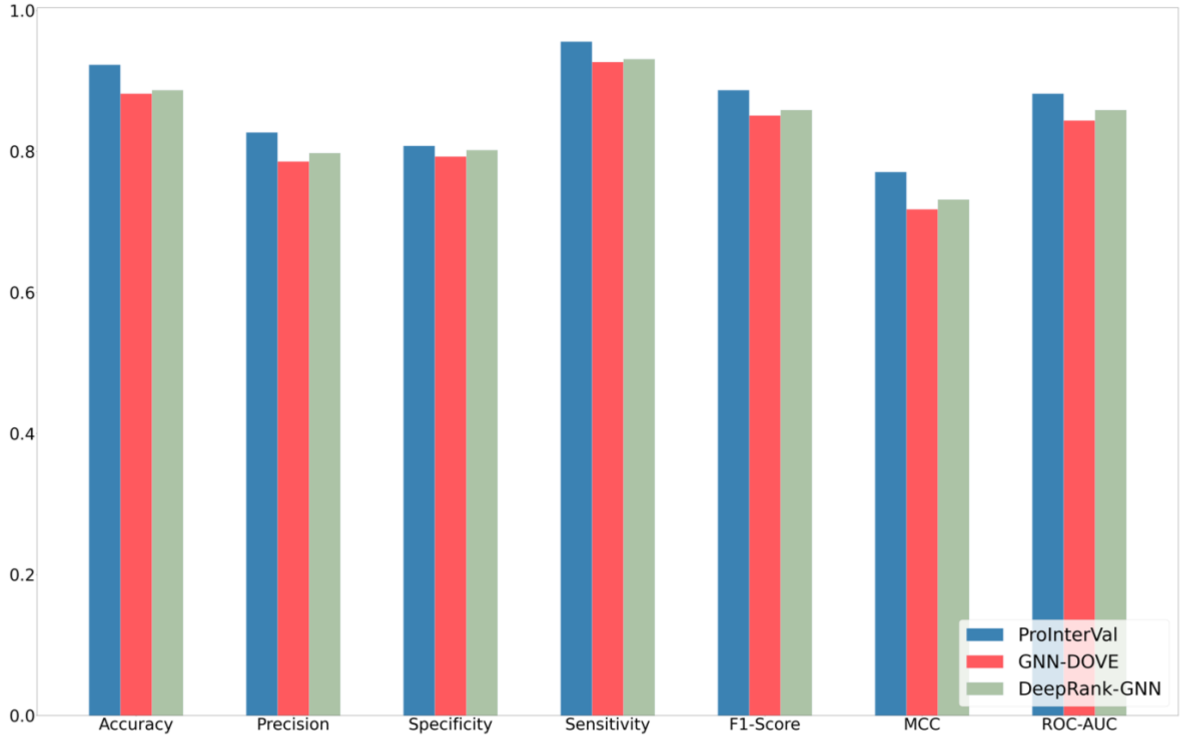

Figure S1: Performance evaluation results of ProInterVal, GNN-DOVE, and DeepRank-GNN on the DeepInterface test set, after retraining the models on the training set of DeepInterface dataset.

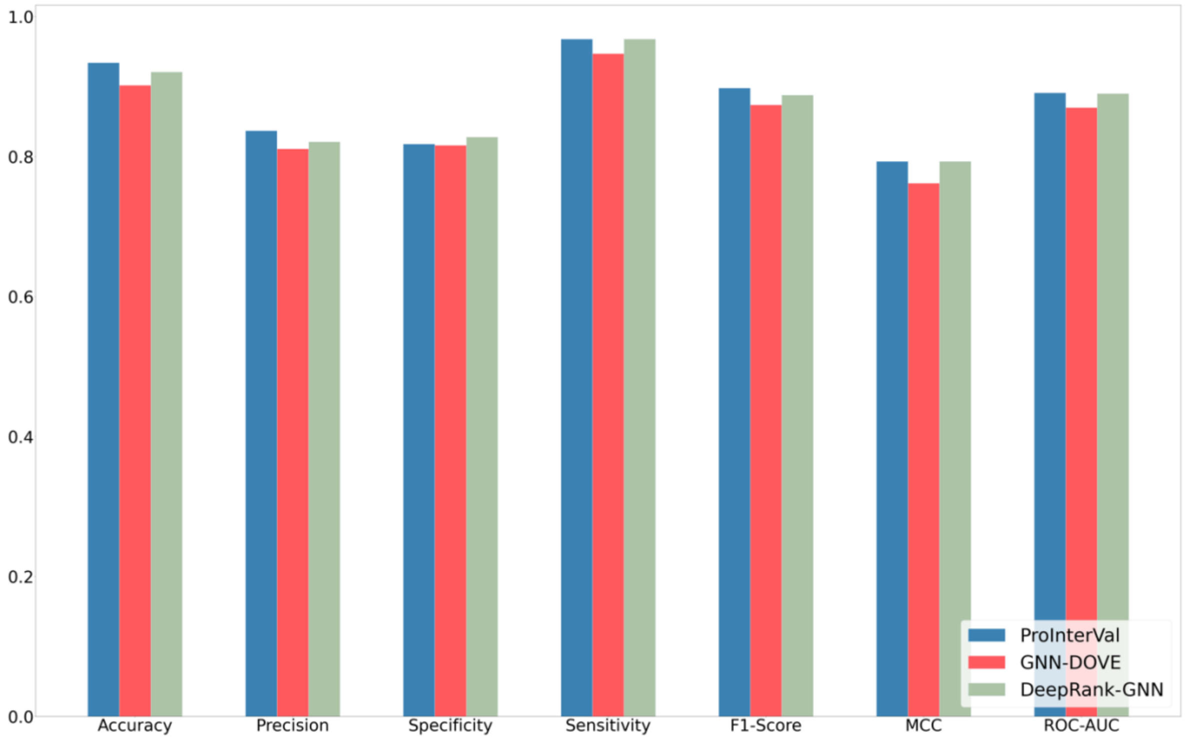

Figure S2: Performance evaluation results of ProInterVal, GNN-DOVE, and DeepRank-GNN on the GNN-DOVE test set, after retraining the models on the training set of GNN-DOVE dataset.

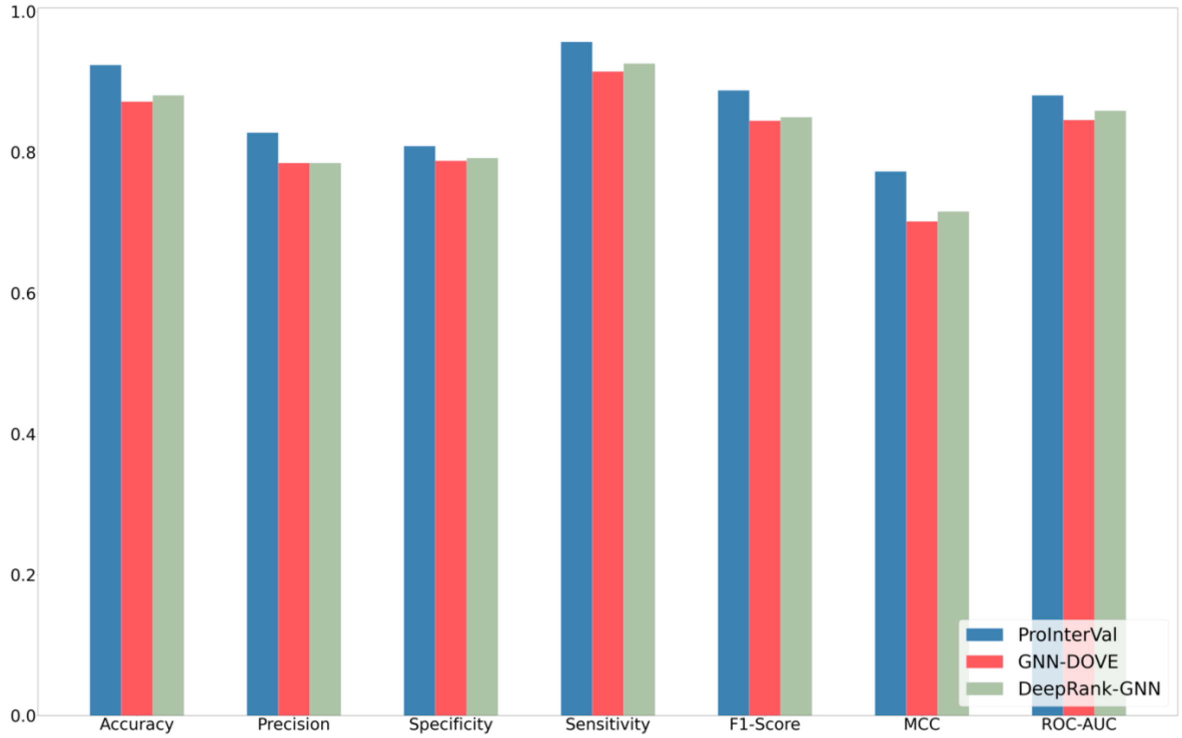

Figure S3: Performance evaluation results of ProInterVal, GNN-DOVE, and DeepRank-GNN on the DeepRank-GNN test set, after retraining the models on the training set of DeepRank-GNN dataset.

REFERENCE

[1] Marzella, DF; Ambrosetti, F; Bonvin, AMJJ; Geng, C; Georgievska, S; Renaud, N; Ridder, L; Xue, LC. 2021. "Protein-protein structures.", SBGrid Data Bank, V1, https://doi.org/10.15785/SBGRID/843.
